# Evaluation of the age-specific relationship between PTH and vitamin D metabolites

**DOI:** 10.1016/j.bonr.2024.101800

**Published:** 2024-08-26

**Authors:** Alexandra Povaliaeva, Artem Zhukov, Viktor Bogdanov, Axenia Bondarenko, Oleg Senko, Anna Kuznetsova, Maxim Kodryan, Vitaliy Ioutsi, Ekaterina Pigarova, Liudmila Rozhinskaya, Natalia Mokrysheva

**Affiliations:** a Endocrinology Research Centre 11, Dmitriya Ul'yanova street, Moscow 117292, Russia; b Life Sciences Research Center, Moscow Institute of Physics and Technology, Dolgoprudniy, Russia; c Federal Research Center "Computer Science and Control" of the Russian Academy of Sciences, Moscow, Russia; d Emanuel Institute of Biochemical Physics of Russian Academy of Sciences, Moscow, Russia; e HSE University, Moscow, Russia

**Keywords:** Ageing, Vitamin D, Vitamin D metabolite ratio, Vitamin D deficiency, Parathyroid hormone, Mass spectrometry

## Abstract

A commonly used method for determining vitamin D sufficiency is the suppression of excess PTH secretion. Conventionally, the main circulating vitamin D metabolite 25(OH)D is used for this assessment, however, the cut-off data for this parameter vary widely in the literature. The role of other metabolites as markers of vitamin D status is actively debated. The aim of our study was to assess the relationship between PTH, age and parameters characterizing vitamin D status, both “classical” – 25(OH)D_3_, and “non-classical” – 24,25(OH)_2_D_3_ and 25(OH)D_3_/24,25(OH)_2_D_3_ (vitamin D metabolite ratio, VMR). This prospective non-controlled cohort study included 162 apparently healthy Caucasian adult volunteers. When PTH was binarized according to the median value, at VMR < 14.9, 25(OH)D_3_ > 9.7 ng/mL and 24,25(OH)_2_D_3_ > 0.64 ng/mL there was a pronounced relationship between PTH and age (*p* = 0.001, *p* = 0.023 and *p* = 0.0134 respectively), with the prevalence of higher PTH levels in older individuals and vice versa. Moreover, at an age of <40.3 years, there was a pronounced relationship between PTH and VMR (*p* < 0.001), and similarly at an age of <54.5 years, there was a pronounced relationship between PTH and 25(OH)D_3_ (*p* = 0.002) as well as between PTH and 24,25(OH)_2_D_3_ (*p* = 0.0038): in younger people, higher PTH values prevailed only in the range of vitamin D insufficiency, while in the older age group this relationship was not demonstrated and PTH values were in general above the median. VMR controlled the correlation between PTH and age more strongly than metabolites 25(OH)D_3_ and 24,25(OH)_2_D_3_ (*p* = 0.0012 vs. *p* > 0.05 and *p* = 0.0385 respectively). The optimal threshold was found equal to 11.7 for VMR such that the relationship between PTH and age in the subset of participants with VMR < 11.7 was characterized by a correlation coefficient of ρ = 0.68 (*p* < 0.001), while the cohort with VMR > 11.7 was characterized by a very weak correlation coefficient of ρ = 0.12 (*p* = 0.218), which is non-significant. In summary, our findings suggest that the relationship between PTH and vitamin D is age-dependent, with a greater susceptibility to elevated PTH among older individuals even with preserved renal function, likely due to the resistance to vitamin D function. We propose VMR can be considered as a potential marker of vitamin D status. These findings require confirmation in larger population-based studies.

## Highlights

• The relationship between PTH and vitamin D is age-dependent.
• VMR controlled the correlation between PTH and age stronger than 25(OH)D_3_ and 24,25(OH)_2_D_3_.
• VMR can be considered as a potential marker of vitamin D status.

## 1 Introduction

The main biological role of vitamin D is the absorption of dietary calcium. One of the most commonly used criteria in determining vitamin D sufficiency is the suppression of the excess parathyroid hormone (PTH) secretion, since the increase in PTH concentration reflects compensation for the inadequate absorption of calcium in the intestine in the state of vitamin D insufficiency. Conventionally, the main circulating vitamin D metabolite 25(OH)D is used for this assessment, however, the cut-off data for this parameter vary widely in the literature (Sai et al., 2011). The role of other metabolites as markers of vitamin D status is actively debated, but their relationship with PTH has been studied to a much lesser extent.

Under the conditions of the vitamin D deficiency, the synthesis of the major predominantly inactive metabolite (24,25(OH)_2_D) is minimized in favor of producing sufficient amounts of the active form of vitamin D (1,25(OH)_2_D) and maintaining adequate calcium absorption in the intestine, as was first shown by Tanaka and DeLuca (Tanaka and DeLuca, 1981). In severe vitamin D deficiency (defined as 25(OH)D < 10 ng/mL), the concentration of 24,25(OH)_2_D becomes undetectable (Kaufmann et al., 2014), while a significant decrease in the percentage of individuals with 24,25(OH)_2_D levels below the detection threshold is observed with 25(OH)D values >20 ng/mL (Cavalier et al., 2020).

It should be noted that very low serum concentrations of 24,25(OH)_2_D may be observed due to a number of reasons, among which the differential diagnosis between vitamin D deficiency and an inactivating mutation of CYP24A1 (the enzyme responsible for the synthesis of 24,25(OH)_2_D from 25(OH)D). To solve this problem, the calculation of the ratio of vitamin D metabolites (25(OH)D/24,25(OH)_2_D, vitamin D metabolite ratio, VMR) is proposed. Under normal conditions VMR is inversely correlated with 25(OH)D and is described by an exponential relationship, while 24,25(OH)_2_D has a strong positive correlation and linear relationship with 25(OH)D (Jones and Kaufmann, 2021).

The relationship between vitamin D and PTH may be influenced by various external and internal factors, with age being one of the prominent candidates. Older age is widely considered to be a risk factor for vitamin D deficiency (Holick et al., 2011; Dedov et al., 2021), which is explained primarily by decreased cutaneous synthesis (Wacker and Holick, 2013). On the other hand, a decrease in renal function, being common in older people, leads to a decrease in the synthesis of 1,25(OH)_2_D and the consumption of 25(OH)D correspondingly (Dusso et al., 2011). In addition, it has long been established that calcium absorption decreases with age (Nordin et al., 1970). Some previously conducted studies aimed at clarifying the effect of age on the relationship between PTH and vitamin D, but they were mainly focused on 25(OH)D and 1,25(OH)_2_D (Vieth et al., 2003; Valcour et al., 2012).

The aim of this study was to assess the relationship between PTH, age and parameters characterizing vitamin D status (both “classical” – 25(OH)D_3_, and “non-classical” – 24,25(OH)_2_D_3_ and VMR).

## 2 Materials and methods

### 2.1 Study population and design

This was a prospective non-controlled cohort study. The study group included 162 apparently healthy Caucasian adult volunteers without a history of metabolic skeletal diseases (including osteoporosis) or disorders of calcium‑phosphorus homeostasis. The exclusion criteria were: vitamin D supplementation or therapy that is presumably associated with alterations in vitamin D metabolism in the three month period prior to the study; severe obesity (body mass index (BMI) >35 kg/m^2^); pregnancy; the presence of granulomatous disease, malabsorption syndrome, liver failure, or chronic kidney disease. All participants were recruited in the period from July 2019 to June 2023. Serum samples were either transferred directly to the laboratory for biochemical analyzes and PTH measurement or were stored at −80 °C avoiding repeated freeze-thaw cycles for measurement of vitamin D metabolites at a later date. The study protocol was approved by the Ethics Committee of Endocrinology Research Centre, Moscow, Russia, on April 10, 2019 (abstract of record No. 6), all participants signed an informed consent to participate in the study.

### 2.2 Laboratory measurements

The serum vitamin D metabolites levels (25(OH)D_3_, 25(OH)D_2_ and 24,25(OH)_2_D_3_) were determined by ultra-high performance liquid chromatography in combination with tandem mass spectrometry (UPLC-MS/MS) using an in-house developed method, described earlier for 2019–2022 (Povaliaeva et al., 2020) and 2022–2023 (Zhukov et al., 2022; Usoltseva et al., 2023). With this technique, the laboratory participates in the DEQAS quality assurance program (lab code 2388) and the results fall within the target range for the analysis of 25(OH)D and 1,25(OH)_2_D metabolites in human serum. Proficiency certificates for the determination of 25(OH)D were issued annually from 2020 to 2024.

PTH levels were evaluated by electrochemiluminescence immunoassay (ELECSYS, Roche, Switzerland; reference range for this and subsequent laboratory parameters are given in the Results section for easier reading). Biochemical parameters of blood serum were assessed by ARCHITECT c8000 analyzer (Abbott, Illinois, United States) using reagents from the same manufacturer according to standard methods. Calibration and quality control were performed in accordance with the manufacturers' recommendations.

### 2.3 Other measurements

At the baseline visit, participants provided medical history via a questionnaire. Serving of dairy products was defined as 100 g of cottage cheese, 200 mL of milk, 125 g of yogurt or 30 g of cheese. Participants' weight was measured in light indoor clothing with a medical scale to the nearest 100 g, and their height was measured with a wall-mounted stadiometer to the nearest centimeter. Body mass index (BMI) was calculated as weight in kilograms divided by height in meters squared. The glomerular filtration rate was calculated according to the recommendations of the NKF-ASN Task Force 2021 (Delgado et al., 2022).

### 2.4 Statistical analysis

Statistical analysis was performed using Statistica version 13.0 (StatSoft, Oklahoma, United States), Data Master (Azforus, Moscow, Russia) and Python statistics and graphics modules. The statistical techniques were aimed to assess the nonlinear relationship of factors. Significance assessment was based on nonparametric permutation test and the Occam's razor principle. The procedure for statistical calculations is described in details in the Appendix. Continuous variables were summarized as median and interquartile range (IQR), binary variables were presented as numbers and percentages. A *p*-value of <0.05 was considered statistically significant.

## 3 Results

The study group consisted predominantly of young women characterized by a moderately healthy lifestyle and suboptimal calcium intake (Table 1).

**Table 1:** General characteristics of the study group.

Parameter	Value (n = 162)
Age (years), median (IQR)	26.2 (24.9; 42.5)
Sex (female/male), n (%)	125(77 %)/37(23 %)
BMI (kg/m^2^), median (IQR)	22.5 (20.0; 26.3)
Current smokers, n (%)	26 (16 %)
Former smokers, n (%)	21 (13 %)
Dairy products consumption (servings per day), median (IQR)	1 (1; 2)
Alcohol consumption (units per week), median (IQR)	0.5 (0; 1)
Exercises lasting >30 min per week, median (IQR)	3 (2; 5)
Number of medications, median (IQR)	0 (0; 1)

Abbreviations: BMI, body mass index; IQR, interquartile range.

25(OH)D_3_ levels <20 ng/mL were observed in half of the study group, and increased PTH was observed in 15 participants (9 %) (Table 2). Only one patient had eGFR below 60 mL/min/1.73m^2^ (57 mL/min/1.73m^2^, new-onset). We observed no significant alterations in calcium and phosphorus serum levels.

**Table 2:** Laboratory parameters of the study group.

Parameter	Value (n = 162)	Reference interval
PTH, pg/mL	37.0 (28.9; 47.2)	15–65 a
25(OH)D_3_, ng/mL	20.9 (13.6; 28.1)	30–100 b
24,25(OH)_2_D_3_, ng/mL	1.6 (0.8; 2.5)	0.5–5.6 c
25(OH)D_3_/24,25(OH)_2_D_3_	13.7 (11.0; 17.5)	7–23 c
Albumin-adjusted calcium, mmol/L	2.27 (2.22; 2.31)	2.15–2.55 a
Phosphorus, mmol/L	1.16 (1.06; 1.27)	0.74–1.52 a
Creatinine, μmol/L	69.8 (65.2; 75.1)	63–110 (male), 50–98 (female) a
eGFR, mL/min/1.73m^2^	106 (97; 126)	–

Abbreviations: PTH, parathyroid hormone; eGFR, estimated glomerular filtration rate.

a Reference ranges are specified according to kit manufacturers' recommendations.

b Reference range is given for total 25(OH)D according to the clinical guidelines (Holick et al., 2011; Dedov et al., 2021); the 25(OH)D_2_ fraction is negligible (<0.5 ng/mL in absolute values) for the purposes of this study.

c Reference ranges are given according to the literature data (Dirks et al., 2016; Tang et al., 2017).

**Fig. 1:**
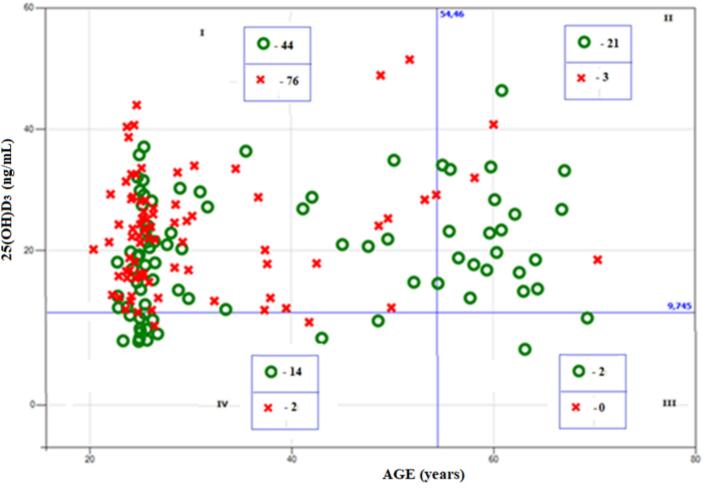
Association of PTH with the combination of age and 25(OH)D_3_. Red crosses correspond to PTH_B = 0 (lower than median value); green circles correspond to PTH_B = 1 (higher than median value).

**Fig. 2:**
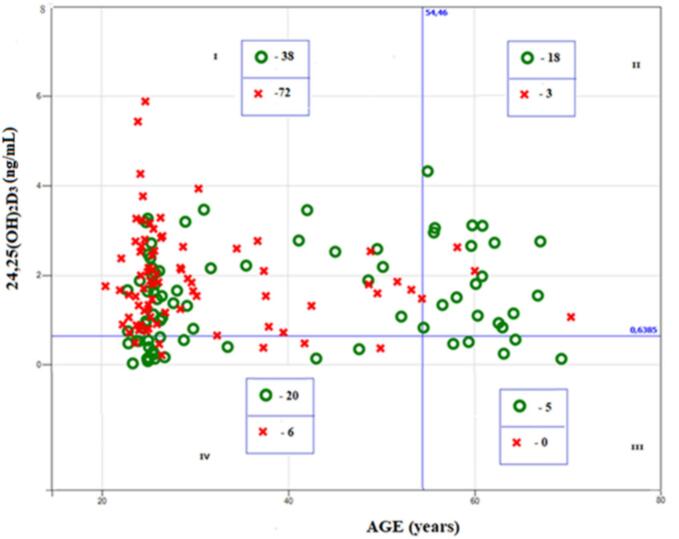
Association of PTH with the combination of age and 24,25(OH)_2_D_3_. Red crosses correspond to PTH_B = 0 (lower than median value); green circles correspond to PTH_B = 1 (higher than median value).

**Fig. 3:**
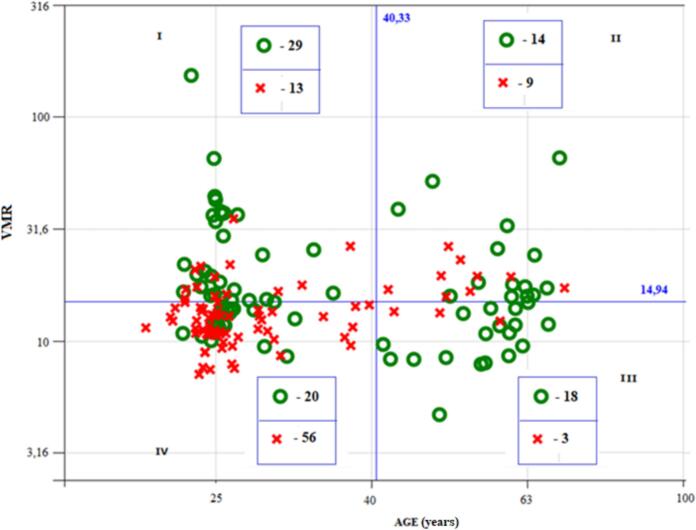
Association of PTH with the combination of age and vitamin D metabolite ratio (VMR). Red crosses correspond to PTH_B = 0 (lower than median value); green circles correspond to PTH_B = 1 (higher than median value). Presented on a logarithmic scale.

At the first stage the relationship between PTH and a combination of age and vitamin D status was studied using optimal valid partitioning (OVP) (Kuznetsova et al., 2014) technique described in Appendix. PTH was binarized in the following way: PTH_B = 0 (lower than median value) and PTH_B = 1 (higher than median value). The aim of performed OVP analysis was to separate in the best way groups with PTH_B = 1 and PTH_B = 0 by age and vitamin D status parameters and to evaluate the significance of this separation. Significance was evaluated using permutation test (Pesarin and Salmaso, 2010) with the number of permutations equal to 5000 and Occam's razor principle (Senko et al., 2022). The results of this analysis when vitamin D status is described by its metabolites 25(OH)D_3_, 24,25(OH)_2_D_3_, as well as VMR are presented in Fig. 1, Fig. 2, Fig. 3, respectively.

It is seen from Fig. 1 that at 25(OH)D_3_ > 9.7 ng/mL there was a pronounced relationship between PTH_B and age. In the cases of age <54.5 years (quadrant I), PTH_B = 0 predominated: 76 cases of PTH_B = 0 versus 44 cases with PTH_B = 1. In the subset of participants with age >54.5 years (quadrant II), cases with PTH_B = 1 predominated: 21 cases of PTH_B = 1 versus 3 cases with PTH_B = 0.

On the contrary, at an age of <54.5 years there was a pronounced relationship between PTH_B and 25(OH)D_3_: in the area of 25(OH)D_3_ < 9.7 (quadrant IV) 14 cases of PTH_B = 1 versus 2 cases with PTH_B = 0, which differed greatly from the proportions in the previously mentioned quadrant I. The significance of the effect for age was estimated at *p* = 0.002, the significance of the effect for 25(OH)D_3_ was estimated at *p* = 0.023.

A similar layout was observed for 24,25(OH)_2_D_3_ (Fig. 2). In participants with 24,25(OH)_2_D_3_ > 0.64 there was a strong association between PTH_B and age. At an age of <54.5 years (quadrant I), cases with PTH_B = 0 predominated: 72 cases of PTH_B = 0 versus 38 cases with PTH_B = 1. At an age >54.5 years (quadrant II), cases with PTH_B = 1 predominated: 18 cases of PTH_B = 1 versus 3 cases with PTH_B = 0.

On the contrary, at an age of <54.5 years, there was a pronounced relationship between PTH_B and 24,25(OH)_2_D_3_: at 24,25(OH)_2_D_3_ < 0.64 (quadrant IV) 20 cases of PTH_B = 1 versus 6 cases with PTH_B = 0, which was markedly different from the proportions in the previously mentioned quadrant I. The significance of the effect for age was estimated at *p* = 0.0038, the significance of the effect for 24,25(OH)_2_D_3_ was estimated at *p* = 0.0134.

A somewhat stronger effect was observed when examining the association of PTH with the combination of age and VMR. It is seen from Fig. 3 that in participants with VMR < 14.9 there was a strong association between PTH_B and age. At an age of <40.3 years (quadrant IV), cases with PTH_B = 0 predominated: 56 cases of PTH_B = 0 versus 20 cases with PTH_B = 1. At an age >40.3 years (quadrant III), cases with PTH_B = 1 predominated: 18 cases of PTH_B = 1 versus 3 cases with PTH_B = 0.

On the contrary, at an age of <40.3 years, there was a pronounced relationship between PTH_B and VMR: at VMR > 14.9 (quadrant I) 29 cases of PTH_B = 1 versus 13 cases with PTH_B = 0, which was markedly different from the proportions in the previously mentioned quadrant IV. The significance of the effect for age was estimated at *p* < 0.001, the significance of the effect for VMR was estimated at *p* = 0.001.

At the second stage nonparametric technique evaluating the significance of the correlation between Y and X in groups that are formed by the third variable Z was used to assess the relationship between PTH, age and vitamin D status. The technique is discussed in Appendix. All *p*-values were calculated using a previously discussed permutation test with 10,000 permutations.

**Fig. 4:**
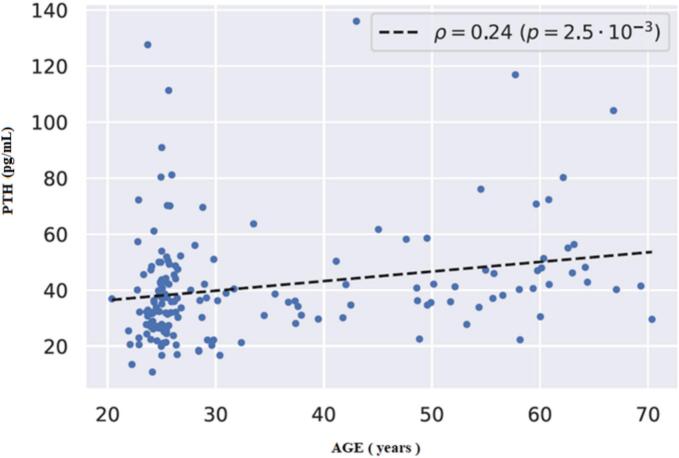
Association between PTH and age in the entire study group (*n* = 162).

The relationship between PTH and age in the entire study group was characterized by a weak positive correlation (*r* = 0.24, *p* = 0.0025), as depicted in Fig. 4.

Next, PTH and age were considered as Y and X variables. In Table 3 p-values $p_{y}$, $p_{x}$ and $p_{z}$ are given when parameters 25(OH)D_3_, 24,25(OH)_2_D_3_ and VMR are used as variable Z.

**Table 3:** Statistical significance of the effect.

	$p_{y}$	$p_{x}$	$p_{z}$
25(OH)D_3_	0.047	0.0689	0.0754
24,25(OH)_2_D_3_	0.0246	0.0349	0.0385
25(OH)D_3_/24,25(OH)_2_D_3_	0.0003	0.0015	0.0012

**Fig. 5:**
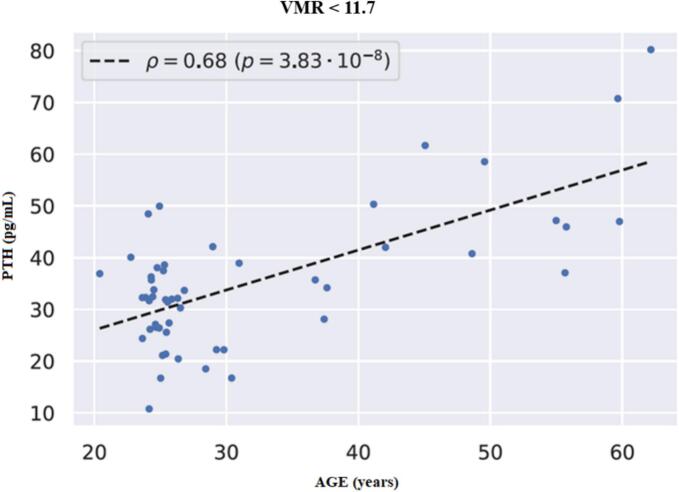
The relationship between PTH and age in the subset of participants with VMR < 11.7 (*n* = 52) was characterized by a correlation coefficient of ρ = 0.68, which is significant at the *p* < 0.001 level according to the standard Student's test.

It is seen from the table that the effect is significant at *p* = 0.0012 and at *p* = 0.0385 when VMR and 24,25(OH)_2_D_3_ are used respectively as variable Z. The effect is not significant at *p* < 0.05 when 25(OH)D_3_ is used as Z. Thus, VMR controls the correlation between PTH and age more strongly than the metabolites 25(OH)D_3_ and 24,25(OH)_2_D_3_. The optimal threshold for VMR was found equal to 11.7. The linear dependence between PTH and age in the sub-set of 52 cases with VMR < 11.7 is seen from Fig. 5.

**Fig. 6:**
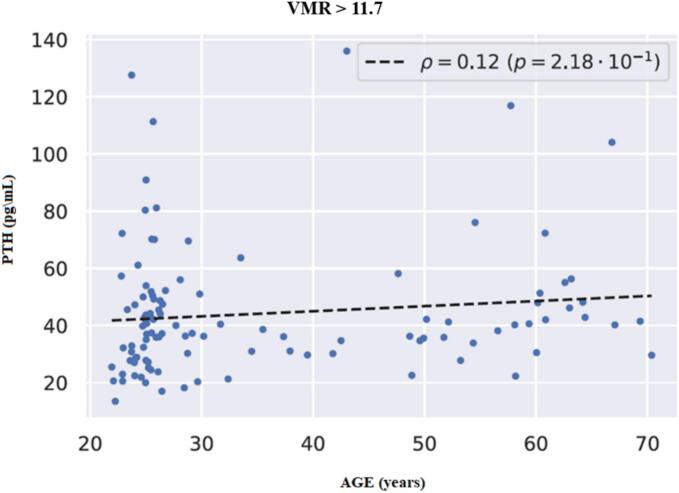
The relationship between PTH and age in the subset of participants with VMR > 11.7 (*n* = 110) was characterized by a very weak correlation coefficient of ρ = 0.12 (*p* = 0.218), which is non-significant.

In Fig. 6, there is no significant association between age and PTH in the sub-set of 110 cases with VMR > 11.7.

## 4 Discussion

This is an exploratory study aimed at the principles of the relationship between PTH and vitamin D. The results suggest that at higher values of 24,25(OH)_2_D_3_ and lower values of VMR (which includes the range of vitamin D sufficiency), there was an association between PTH and age such that higher PTH values predominated in older individuals and vice versa. With values of 24,25(OH)_2_D_3_ and VMR in the opposite range of the axis (including severe vitamin D deficiency), such a relationship was absent and PTH values above the median predominated at all ages. These findings are supported by the similar pattern observed for the conventional metabolite defining vitamin D status – 25(OH)D_3_. Moreover, the influence of age was noted when analyzing the relationship between PTH and parameters of vitamin D metabolism: in younger people, higher PTH values prevailed only in the range of vitamin D insufficiency, while in the older age subgroups this relationship was not demonstrated and PTH values were in general above the median.

Furthermore, we have demonstrated a different nature of the relationship between PTH and age at VMR values less and more than the obtained “cut-off point”, which is close to the lower quartile. With VMR values <11.7 (characteristic of the normal range of 25(OH)D_3_ values and vitamin D sufficiency respectively), older patients were characterized by higher PTH values. This is consistent with the literature showing that older individuals have higher PTH levels than younger individuals even with comparable 25(OH)D levels (Vieth et al., 2003; Valcour et al., 2012). Our results, along with previous work by other groups, may indicate the need to consider age as an independent risk factor for secondary hyperparathyroidism, and also serve as confirmation of existing recommendations for higher vitamin D intake in older adults (Holick et al., 2011), as preventing PTH increase in them may require higher levels of vitamin D. It should be noted that at higher VMR values, which include the range of vitamin D deficiency, no association of PTH and age was noted, indicating a more complex regulation of PTH production in these conditions. Interestingly, VMR controlled the correlation between PTH and age more strongly than metabolites 25(OH)D_3_ and 24,25(OH)_2_D_3_, which strengthens its high potential as a marker of vitamin D status.

It might be assumed that the increased impact of the vitamin D status on serum PTH levels in older populations may reflect decreased calcium absorption and/or renal function observed with age. It seems unlikely to us that the results we observed could be associated with reduced production of the active metabolite 1,25(OH)_2_D_3_, since the included individuals had preserved renal function, while the production of 1,25(OH)_2_D_3_ appears to be determined primarily by the functional state of the kidneys, and not by the increasing age itself (Vieth et al., 2003). The hypothesis of relative resistance to the action of 1,25(OH)_2_D_3_ in older individuals seems more likely to apply (Eastell et al., 1991; Pattanaungkul et al., 2015).

Our cohort was relatively small with a predominance of young people, and therefore it seems not feasible to project the obtained results to the general population. In addition, it was characterized by suboptimal dietary calcium intake, which may have affected the results. Other limitations of the study include the following: key parameters of calcium‑phosphorus homeostasis (1,25(OH)_2_D, fibroblast growth factor-23, ionized calcium, magnesium) were not taken into account; a single measure of PTH is not optimal due to biologic variation especially in this population with suboptimal calcium intake; and the participants were not screened for other abnormalities (such as elevated serum transaminase, hyperglycemia, clotting factor, low BMD, etc). However, our data provide an important direction for future research, since an elevated PTH is associated with both bone health deterioration (Sahota et al., 2001; Sahota et al., 2004; Qu et al., 2020) and non-skeletal consequences (Yang et al., 2016; Anderson et al., 2011; Grandi et al., 2011; Wu et al., 2023) even out of context of primary hyperparathyroidism. A better understanding of the dynamics of the relationship between PTH and vitamin D and the factors influencing this relationship is an important step towards improving clinical approaches.

## 5 Conclusions

The relationship between PTH and vitamin D is age-dependent, with a greater susceptibility to elevated PTH among older individuals even with preserved renal function, likely due to resistance to vitamin D function. Interestingly, VMR controlled the correlation between PTH and age more strongly than metabolites 25(OH)D_3_ and 24,25(OH)_2_D_3_, which strengthens its high potential as a marker of vitamin D status. The findings require confirmation in larger population-based studies.

## Funding

This research was funded by the 10.13039/501100006769Russian Science Foundation, grant number 19-15-00243-P.

## Institutional review board statement

This study was performed in line with the principles of the Declaration of Helsinki. Approval was granted by the Ethics Committee of Endocrinology Research Centre, Moscow, Russia on April 10, 2019 (abstract of record No. 6). Informed consent was obtained from all individual participants included in the study.

## Informed consent statement

Informed consent was obtained from all subjects involved in the study.

## CRediT authorship contribution statement

**Alexandra Povaliaeva:** Writing – original draft, Formal analysis, Conceptualization. **Artem Zhukov:** Writing – review & editing, Project administration, Conceptualization. **Viktor Bogdanov:** Writing – review & editing, Investigation. **Axenia Bondarenko:** Writing – review & editing, Data curation. **Oleg Senko:** Writing – review & editing, Visualization, Resources, Formal analysis. **Anna Kuznetsova:** Visualization, Software, Formal analysis. **Maxim Kodryan:** Visualization, Software, Formal analysis. **Vitaliy Ioutsi:** Investigation. **Ekaterina Pigarova:** Writing – review & editing, Supervision, Conceptualization. **Liudmila Rozhinskaya:** Writing – review & editing, Supervision, Resources, Funding acquisition, Conceptualization. **Natalia Mokrysheva:** Supervision, Resources, Funding acquisition, Conceptualization.

## Declaration of competing interest

The authors have no relevant financial or non-financial interests to disclose.

## Data Availability

Data will be made available on request.

## Appendix A

### A.1 Optimal partitioning method

The goal is to assess dependence of binary $Y\in \left\{01\right\}$ on explanatory variables $X^{\prime }$ and $X^{"}$ by dataset $S=\left\{\left(y_{1},x\prime_{1},x{"}_{1}\;\right),\ldots ,\left(y_{m},x\prime_{m},x{"}_{m}\right)\right\}.$The search for optimal partitions presented in the Fig. 1, Fig. 2, Fig. 3 is carried out by dataset within a family of partitions of the ranges of values of two explanatory variables into four subareas (quadrants) using boundaries parallel to the coordinate axes. The goal is to find partition that in the best way separates two compared groups. The quality of partition with k quadrants $q_{1},\ldots ,q_{k}$ is described by functional $Q\left(S\right)=\sum_{i=1}^{k}{\left(\nu_{i}-\nu_{0}\right)}^{2}m_{i}$, where $\nu_{0}$ is fraction of objects with $y_{j}=1$ from dataset S, $\nu_{i}$ is fraction of objects with $y_{j}=1$ from dataset S inside quadrant $q_{i}$, $m_{l}$ is number of objects 1 from dataset S inside quadrant $q_{i}$. So the search of partition where $Q\left(S\right)$ is maximal is implemented. Let $b\prime_{o}$, $b{"}_{o}$ are boundaries for variables $X^{\prime }$ and $X^{"}$ and $Q_{m}\left(S\right)$ is $Q\left(S\right)$ value for optimal partition. Statistical significance of found in such a way regularities is assessed using a permutation test and variant of Occam's razor principle (Senko et al., 2022) stating that more complicated may be used only if it allows reject null hypothesis about exhaustive description of studied dependence by simple model. Statistical significance of contributions of $X^{\prime }$ and $X^{"}$ to two-dimensional regularity are evaluated separately. To evaluate statistical significance of $X^{\prime }$ contribution null hypothesis ${H^{”}}_{0}$ that dependence Y from $X^{\prime }$ and $X^{"}$ is exhaustively described by one variable $X^{"}\;$only. In other words, $H{"}_{0}$ assumes independence of Y from $X^{\prime }$ and $X^{"}$ inside segments to the left and to the right of boundary $b\prime_{o}$. Evaluating of statistical significance of $X^{\prime }$ contribution is reduced to testing of null hypothesis $H\prime_{0}$ that Y is independent from $X^{\prime }$ and $X^{"}$ inside segments to the left and to the right of boundary $b{"}_{o}$. Let $\tilde{l\;}=\left\{l_{1},\ldots ,l_{\mathrm{ml}}\right\}$ и $\tilde{r\;}=\left\{r_{1},\ldots ,r_{\mathrm{mr}}\right\}$ are the numbers of objects with $X^{"}<b{"}_{o}$ and $X^{"}>b{"}_{o}$ respectively. Modification of permutation is used to reject or accept $H\prime_{0}$. Sets of random permutations $\left\{f_{1}^{l},\ldots ,f_{N}^{l}\right\}$ and $\left\{f_{1}^{r},\ldots ,f_{N}^{r}\right\}$ of natural numbers from sets $\tilde{l\;}$ and $\tilde{r\;}$ are calculated using a random number generator. Sets $S_{j}^{\mathrm{pl}}=\left\{\left(x_{f_{j}^{l}\left(l_{1}\right)},y_{1},z_{1}\right),\ldots ,\left(x_{f_{j}^{l}\left(l_{\mathrm{ml}}\right)},y_{m},z_{m}\right)\right\}$ and $S_{j}^{\mathrm{pr}}=\left\{\left(x_{f_{j}^{r}\left(r_{1}\right)},y_{1},z_{1}\right),\ldots ,\left(x_{f_{j}^{r}\left(r_{\mathrm{ml}}\right)},y_{m},z_{m}\right)\right\}$ are received by permutations $f_{j}^{l}$ and $f_{j}^{r}$. Let $S_{j}^{p}=S_{j}^{\mathrm{pl}}\cup S_{j}^{\mathrm{pr}}$. Statistical significance of $X^{\prime }$ contribution is evaluated with the help of *p*-value calculated from set of random samples $\left\{S_{j}^{p}|j=1,\ldots ,N\right\}$ by formula $p=\frac{\left|\{Q_{m}(S_{j}^{p}\right)>Q_{m}\left(S\right)|j=1,\ldots ,N\}|}{N}$. Statistical significance of $X^{"}$ contribution is evaluated in the same way but sets $\tilde{l\;}$ and $\tilde{r\;}$ are calculated by $X^{\prime }$ using boundary $b\prime_{o}.$

### A.2 Influence of the third factor on the correlation of two variables

The technique is based on well-known permutation test (Pesarin and Salmaso, 2010). Groups are formed according to a simple rule with threshold δ: object is put to left group if Z<δ and object is put to right group if Z>δ. The optimal threshold δ is searched through maximizing the special functional q. The assessment of the statistical significance of the effect associated with the difference in correlations in the left and right groups is based on an attempt to refute each of the three null hypotheses $H_{0}^{y}$, $H_{0}^{x}$ and $H_{0}^{z}$: $H_{0}^{y}$ assumes the independence of Y on combination of X and Z; $H_{0}^{x}$ assumes the independence of X on combination of Y and Z; $H_{0}^{z}$ assumes the independence of Z on combination of X and Y. Each hypothesis is tested by comparing test statistic F at initial dataset S with F value at random samples $S_{1}^{r},\ldots ,S_{N}^{r}$ which are generated from initial dataset S by random permutations of one of the three variables X,Yand Z relative to the fixed positions of the other two variables. To reject or accept $H_{0}^{x}$, a set of random permutations $\left\{f_{1},\ldots ,f_{N}\right\}$ of natural numbers from the set $\left\{1,\ldots ,m\right\}$ is calculated using a random number generator. Random sample $S_{j}^{p}=\left\{\left(x_{f_{j}\left(1\right)},y_{1},z_{1}\right),\ldots ,\left(x_{f_{j}\left(m\right)},y_{m},z_{m}\right)\right\}$ is received from dataset S with the help of random permutation $f_{j}$. Set of random samples $\left\{S_{j}^{p}|j=1,\ldots ,N\right\}$ is used to calculate *p*-value by formula $p_{x}=\frac{\left|\{F(S_{j}^{p}\right)>F\left(S\right)|j=1,\ldots ,N\}|}{N}$. To test null hypotheses $H_{0}^{Y}$ and $H_{0}^{z}$ *p*-values $p_{y}$ and $p_{z}\;$are calculated in the same way as $p_{x}$ but by permuting Y and Z instead of permuting X. Our previous studies have shown that the greatest effectiveness of the criterion is achieved when using test statistic based on the well-known Fisher's z-transform, first proposed by him back in 1915. The reasons for using the criterion presented below are discussed in detail in (Mazilina et al., 2021). Suppose that the sample objects to the left and to the right of the boundary δ for the third factor Z are independently generated from bivariate normal distributions with the correlation coefficients between the variables X and Y equal to $\rho_{l}$ and $\rho_{r}$, respectively. Suppose that to the left of the boundary δ there is a subsample $S_{l}$ of $m_{l}$ objects with the correlation coefficient $r\left(S_{l}\right)$; to the right of the boundary δ there is a subsample $S_{r}$ of $m_{r}$ objects with the correlation coefficient $r\left(S_{r}\right)$. Fisher's transformation for the correlation coefficients $R\left(S_{l}\right)$ and $R\left(S_{r}\right)$ are $z_{l}=\frac{1}{2}\ln\left(\frac{1+R\left(S_{l}\right)}{1-R\left(S_{l}\right)}\right)$ and $z_{r}=\frac{1}{2}\ln\left(\frac{1+R\left(S_{r}\right)}{1-R\left(S_{r}\right)}\right)$ respectively. Variables $z_{l}$ and $z_{r}$ are distributed close to normal with means $\frac{1}{2}\ln\left(\frac{1+\rho_{l}}{1-\rho_{l}}\right)$ and $\frac{1}{2}\ln\left(\frac{1+\rho_{r}}{1-\rho_{r}}\right)$ and standard deviations $\sqrt{m_{l}-3}$ and $\sqrt{m_{r}-3}$ respectively. Variables $Z_{l}=\frac{1}{2}\ln\left(\frac{1+R\left(S_{l}\right)}{1-R\left(S_{l}\right)}\right)\sqrt{m_{l}-3}$ and $Z_{r}=\frac{1}{2}\ln\left(\frac{1+R\left(S_{r}\right)}{1-R\left(S_{r}\right)}\right)\sqrt{m_{r}-3}$ are distributed close to normal $N\left(01\right)$ if null hypothesis $\rho_{l}=\rho_{r}=0$ is true. Let $p_{l}=1-\Phi \left(Z_{l}\right)$ and $p_{r}=1-\Phi \left(Z_{r}\right)$ where Φ is cumulative distribution function of the standard normal distribution. The differences between the coefficients are described using the statistic $q\left(\delta ,S\right)$. The search for the optimal value of δ is carried out within the set B which is subset of all unique boundaries from the set $\left\{\frac{z_{1}^{u}+z_{2}^{u}}{2},\ldots ,\frac{z_{m-1}^{u}+z_{m}^{u}}{2}\right\}$, where $\left\{z_{1}^{u},\ldots ,z_{m}^{u}\right\}$ are variable Z values from S sorted in ascending order. At that δ is searched for which $q\left(\delta ,S\right)$ is maximal. Statistic $-\ln p_{l}+\ln p_{r}\;$may be used as $q\left(\delta ,S\right)$ but in this research the better significance was received when $\left(\delta ,S\right)=\max\left(-\ln p_{l},-\ln p_{r}\right)$. Functional $F\left(S\right)=\max_{\mathrm{\delta \epsilon B}}q\left(\delta ,S\right)$ is used as statistic of permutation test that is discussed previously. It should be noted that despite the use of the standard normal distribution when constructing test statistics, the null hypotheses $H_{0}^{y}$, $H_{0}^{x}$ and $H_{0}^{z}$ do not contain assumptions about the nature of the distributions.
